# Small Molecule Drug C381 Attenuates Brain Vascular Damage Following Repetitive Mild Traumatic Injury

**DOI:** 10.1089/neur.2024.0060

**Published:** 2024-10-22

**Authors:** Lulin Li, Andy Nguyen, Brian Zhao, Ryan Vest, Lakshmi Yerra, Bryan Sun, Jian Luo

**Affiliations:** ^1^ Palo Alto Veterans Institute for Research, VA Palo Alto Health Care System, Palo Alto, California, USA.; ^2^ Department of Chemical Engineering, Stanford University, Stanford, California, USA.; ^3^ Polytrauma System of Care, VA Palo Alto Health Care System, Palo Alto, California, USA.

**Keywords:** mild traumatic brain injury, small molecule, therapy, vascular injury

## Abstract

Traumatic brain injury (TBI) remains a significant public health concern, with no effective therapeutic interventions to ameliorate the enduring consequences. The prevailing understanding of TBI pathophysiology indicates a central role for vascular dysfunction. Transforming growth factor-β (TGF-β) is a multifunctional cytokine crucial for vascular development. Aberrant TGF-β signaling is implicated in vascular pathologies associated with various neurological conditions. We recently developed a novel small molecule drug, C381, a TGF-β activator with the ability to restore lysosomal function. Here we used a mouse model of repetitive mild TBI (mTBI) to examine whether C381 would attenuate vascular injury. We first employed RNA-seq analysis to investigate the gene expression patterns associated with mTBI and evaluated the therapeutic potential of C381 in mitigating these changes. Our results demonstrate distinct mTBI-related gene expression signatures, prominently implicating pathways related to vascular integrity and endothelial function. Notably, treatment with C381 reversed these mTBI-induced gene expression changes. Immunohistochemical analysis further corroborated these findings, revealing that C381 treatment attenuated vascular damage in mTBI-affected brain tissue. These findings strongly support the potential clinical usefulness of C381 as a novel therapeutic intervention for mTBI.

## Introduction

Traumatic brain injury (TBI) stands as a significant contributor to death and disability worldwide. TBI has the highest incidence among all common neurological disorders and thus remains a major global health problem.^1^ The mild form of TBI (mTBI, often referred to as concussion) is the major type and accounts for up to 90% of all TBIs.^1^ Patients experiencing mTBI often exhibit acute symptoms, cognitive dysfunction, and problems in day-to-day functioning that resolve within days to weeks. However, many patients with mTBI do not fully recover from their initial injury and continue to experience persistent symptoms or develop long-term complications.^2^ In recent years, the consequences of repetitive mTBI have garnered significant attention, especially among individuals involved in contact sports or in military operations, where the likelihood of repeated concussions is markedly elevated. Recurrent brain injuries, even if mild in nature, may induce cumulative effects and impede neurological recovery.^3,4^ Therefore, repetitive mTBI has been linked to heightened symptom severity, prolonged recovery periods, and earlier onset of age-related memory deficits and dementia.^5^ Repeated concussions have also been associated with chronic traumatic encephalopathy, a neurodegenerative disorder with progressive impairments of memory and cognition, as well as depression, anxiety, and motor abnormalities.^6–8^ Supporting evidence from animal studies underscores the notion that repetitive mTBI induces more severe pathological features and behavioral deficits compared with a single injury.^9^

The pathophysiology of mTBI is not completely understood and likely involves multiple mechanisms, including axonal damage, neuroinflammation, excitotoxicity, oxidative stress, and neurovascular injury. Neurovascular injury is a critical component of TBI pathology, often occurring concurrently with the primary insult to the brain tissue. When a TBI transpires, the sudden and forceful impact of the acceleration–deceleration forces causes the brain to bounce or rotate inside the skull, resulting in damage to the brain blood vessels and disruption of the blood–brain barrier (BBB). These vascular disruptions exacerbate the initial injury by compromising blood flow and oxygen delivery to vital areas of the brain, causing ischemia, and potentially exacerbating neurological deficits. Additionally, disruption of the BBB and leakage of blood components into surrounding brain tissue can trigger inflammation and oxidative stress,^10,11^ further exacerbating secondary brain damage that impairs the brain’s ability to heal and recover.^12^ Neurovascular injury with disruption of BBB is a common feature of mTBI patients.^12–14^ Mechanistically, mTBI causes dysfunction in the endothelial cells and disrupts their communication with pericytes.^15^ mTBI also results in the upregulation of vascular endothelial growth factor and metallopeptidases.^11^ These events may persist chronically^13,14^ and contribute to reduced cerebral blood flow, tissue hypoxia, and BBB breakdown.^12^ Recently, BBB dysfunction has been recognized as a potential mechanistic link between mTBI and the development of neurodegenerative diseases.^16,17^ The complex interplay between these mechanisms is therefore an active area of research, and understanding the underlying mechanisms of vascular injury and BBB disruption in mTBI may facilitate the development of new treatments for this condition.

Transforming growth factor-β (TGF-β) is a multifunctional cytokine that is involved in regulating cell growth, differentiation, migration, and extracellular matrix production. TGF-β is an important injury response factor in the brain and has broad effects on neurons, glial cells, and endothelial cells.^18,19^ TGF-β signaling in endothelial cells is essential for vascular development and perturbed TGF-β signaling contributes to vascular pathologies in many neurological conditions.^20^ TGF-β signaling stabilizes cerebrovascular endothelial cell–pericyte interactions and strengthens the integrity of the BBB. Mice deficient in TGF-β signaling specifically in endothelial cells show increased BBB permeability, BBB breakdown, and intracranial hemorrhage.^21^ In the context of vascular injury, TGF-β has been shown to be involved in a number of processes, including inflammation, extracellular matrix deposition, and cellular proliferation and migration. Molecular components of TGF-β signaling pathway are dysregulated after TBI and modulation of TGF-β signaling alters pathological and behavioral outcomes of TBI in animal models (reviewed in Luo^22^) TGF-β has been identified as a key factor involved in the pathological aspects of vascular dementia.^23^ Targeting TGF-β signaling in the brain is thought to be a promising therapeutic strategy to mitigate vascular pathology and improve cognitive functions.^23^

To harness the therapeutic potential of augmenting TGF-β signaling, we recently developed a novel small molecule drug, C381.^24^ This molecule effectively activated TGF-β signaling in the central nervous system in a dose-dependent manner. Notably, C381 exhibited potent anti-inflammatory and neuroprotective activity in multiple mouse models of neurodegenerative disease.^25^ Genome-wide CRISPR interference drug target identification screen implicated vacuolar-type H^+^-ATPase, the proton pump responsible for lysosome acidification.^25^ Further functional investigations confirmed that C381 physically interacts with the lysosome, promotes lysosomal acidification, increases breakdown of lysosomal content, and rectify lysosomal defects in the progranulin deficient mice *in vivo*.^25^ Therefore, C381 is a novel small molecule TGF-β activator and has the ability to restore lysosomal function.^24^

Given the significant involvement of vascular injury in the pathophysiology of mTBI, alongside the recognized role of TGF-β signaling in this cascade and the demonstrated efficacy of C381 in activating this pathway, we hypothesized that treatment with C381 would attenuate mTBI-induced brain vascular injury and associated neuropathology. We tested this hypothesis in a mouse model of repetitive mTBI developed in our lab.^26,27^ Previous *in vivo* near-infrared imaging studies have revealed a rapid decline in cerebral blood flow post-mTBI, followed by BBB leakage.^28^ This model thus offers an appropriate platform for testing our hypothesis.

## Materials and Methods

### Mice

All animal studies were approved by the VA Palo Alto IACUC and were performed according to the guidelines outlined in the NIH’s Guide for the Care and Use of Laboratory Animals. All mice (2 months old male, wildtype C57BL/6J) were obtained from The Jackson’s Laboratory (Stock No:000664). All mice were group-housed in a temperature- and light-controlled (12-hour light–dark cycle) environment, with *ad libitum* access to food and water. All animals were randomized for group allocations and surgical procedures. The researchers performing experimental procedures and data analysis were blinded and unaware of group allocations.

### Closed-head model of repetitive mTBI

The procedures of closed head mTBI and sham injury were previously described.^26,27^ Briefly, a benchmark stereotaxic impactor (MyNeurolab, St. Louis, MO) actuator was mounted on a stereotaxic frame (David Kopf Instruments, Tujunga, CA) at a 40° angle with a 5-mm impactor tip. After isoflurane anesthesia induction, mice were placed in a foam mold held in prone position on the stereotaxic frame and maintained under anesthesia for the duration of the procedure. The stereotaxic arm was adjusted so that the head impact was at a fixed point relative to the right eye and ear, corresponding to the S1 somatosensory cortex.^26,27^ The impact delivered by the device to the head was 5.0 m/s with a dwell time of 0.2 sec and impact depth of 5 mm. After impact, the mice were recovered from anesthesia on a warming pad before returning to their home cages. For repetitive injuries, three identical impact procedures were performed at an interval of 24 ± 1 h. For sham injury, the previous procedure was performed except that the impact device was discharged in the air. In this closed head injury model, there are no incisions and no craniotomy.

### C381 treatment

**FIG. 1.**
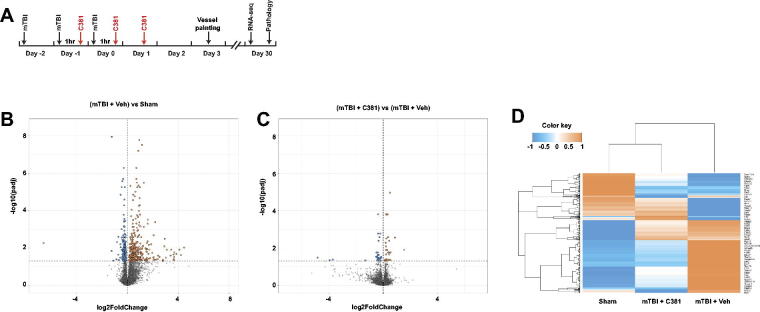
RNA-seq and differential transcriptome analysis. **(A)** Experimental design. **(B–C)** Mice were perfused at 1 month after injury, and their brains were extracted. Ipsilateral hippocampi were further dissected and subjected to RNA-seq analysis. Volcano plot visualizing the number of differentially expressed genes (DEGs) with respect to each comparison. The *y*-axis displays the −log_10_ padj (adjusted *p*-value) for each gene, while the *x*-axis displays the log_2_ fold change (indicated by circle size). Horizontal dotted line indicates the cutoff criteria for DEGs with padj ≤5%. Red circles indicate upregulated genes while blue circles indicate downregulated genes. Black circles represent nondifferentially expressed genes. **(D)** Heatmap of differentially expressed genes (rows) was generated by unsupervised hierarchical clustering and resulted in clustering of the three groups. Red indicates upregulation and blue indicates downregulation.

The compound C381 (10 mg/kg body weight) was formulated in 10% dimethyl sulfoxide (DMSO):15% Solutol:75% sterile water and administered through intraperitoneal injections, as previously described.^25^ The same volume of 10% DMSO:15% Solutol:75% sterile water (without C381) was used as a control treatment. C381 was administered three times: 1 h after the second, third impact and 24 h after the third impact (Fig. 1A).

### Vessel painting and imaging

Mice were deeply anesthetized and perfused via the left ventricle with 10 ml of DiI/liposome solution followed by 10 ml of 4% paraformaldehyde (5 ml/min).^29^ The mouse brain was isolated and post-fixed in the same fixative overnight at 4°C. Macroscopic images of the fixed brains were captured using wide-field fluorescence microscopy (BZ-X700, Keyence Corp, Osaka, Japan).^30^ To obtain whole-brain axial images from a dorsal view, the brain was carefully positioned between two glass slides. Gentle pressure was applied to slightly flatten the dorsal surface so that the entire cortical vasculature of the cerebrum was imaged. Z-stack images of the surface cortical vessels were acquired at 2× magnification with a 1-mm depth of field. Images of the entire brain were reconstructed using the XY-stitching and Z-stack features of BZ-X Analyzer software (Keyence Corp, Osaka, Japan). Image analysis was performed to quantify a region of interest encompassing the lesion area (vessel loss/disruption) with ImageJ.

### Tissue processing

To prepare the mouse brains for histological analysis, mice were perfused transcardially with saline, and their brains were extracted and fixed for 48 h in 4% paraformaldehyde and later equilibrated in 30% sucrose.^26,27^ The brains were coronally sectioned a thickness of 40 µm with a sliding microtome (SM2010 R, Leica, Allendale, NJ). The sections from the sampling region (Fig. 1A) were serially collected in 12 tubes and stored in cryoprotective medium.

### Immunohistochemistry

Immunohistochemistry was performed on free-floating sections following standard procedures.^26,27^ The sample region in this study (Fig. 1A) covers the impact epicenter and surrounding tissue, roughly from 0.98 to −2.06 mm to Bregma.^27^ This resulted typically in 6–7 sections/tube (sections collected in 12 tubes) separated roughly by 12 × 40 µm. For each staining all sections from one tube were used. Free-floating sections were permeabilized, blocked, and stained overnight at 4°C with the following primary antibodies at the designated concentrations: GFAP (1:1000; Catalogue #: M0761; Agilent, Santa Clara, CA), C3 (1:1000; Catalogue #: 06–519; MilliporeSigma, Burlington, MA), and calbindin (1:10000, Catalogue #: CB38, Burgdorf, Switzerland). Sections were washed, stained with Alexa Fluor-conjugated secondary antibodies (1:250) and mounted under a coverslip. Images covering the whole hippocampus (10×, for GFAP and C3) or CA1 (20×, for calbindin) were acquired through on a confocal laser-scanning microscope (Zeiss LSM880, Carl Zeiss Microscopy, White Plains, NY). Image analysis of immunoreactivity was performed using NIH ImageJ software (Bethesda, MD) by a blinded observer. The images were first converted to 8-bit grey-scale images and then converted into binary positive/negative images by thresholding held constantly for all images in a given brain region. Percent area fraction covered by the threshold was determined by ImageJ. The average of values obtained from all sections was used for each animal for statistical analysis.

### Data and statistical analysis

Data are reported as mean ± standard deviation (SD). Statistical analysis was conducted with Prism software (version 10) (GraphPad Software, La Jolla, CA) by analysis of variance (ANOVA) for multiple groups. Appropriate *post hoc* tests (recommended by Prism) were used to compare pairs of groups following ANOVA. *p*-Value <0.05 was considered statistically significant.

### RNA-seq

In a separate experiment, mice were perfused 1 month after injury (and C381 treatment), and their brains were extracted. Ipsilateral hippocampi were further dissected and subjected to RNA-seq analysis.^26^ Briefly, total RNA was extracted using RNeasy Mini Kit (Qiagen, Germantown, MD) per the manufacturer’s instructions. The concentration of RNA was measured using a NanoDrop 8000 UV-Vis Spectrophotometer (Thermo Fisher Scientific, Waltham, MA). RNA integrity number (RIN) was measured using the Agilent Bioanalyzer (Agilent Technologies, Santa Clara, CA). Total RNA samples with RIN >9.0 and a concentration of 150 ng/μL or higher were used for RNA sequencing. Sequence libraries were generated and sequenced by Novogene (Sacramento, CA). Insert size of 250–300 bp was used for cDNA library preparation. Libraries were sequenced on the Illumina platform for 150-bp paired-end reads. Reference genome and annotation files were downloaded from Ensembl, and RNA-seq data were aligned to the reference genome using the Spliced Transcripts Alignment to a Reference software. HTSeq was used to obtain read counts of the mapped genes, and the DESeq2 package was used for differential expression analysis (https://rpubs.com/ge600/deseq2). *p*-Values were adjusted by the Benjamini–Hochberg method, with padj (adjusted *p*-value) <0.05 considered as significantly differential expression. Functional enrichment analyses including Gene Ontology (GO) term and pathway analysis was performed through a web-based tool Enrichr (https://maayanlab.cloud/Enrichr/).

## Results

### RNA-seq analysis unveils a pivotal role of vascular damage in the pathophysiology of mTBI and the reversal effects of C381 treatment on gene expression

To identify genes and pathways that may be involved in mTBI-associated pathology, we performed RNA-seq analysis, using total RNA isolated from the mouse hippocampus (1 month after injury). Through differential expression analysis of RNA-seq data, we identified 501 mTBI-induced, differentially expressed genes (DEGs) (*padj* < 0.05) by comparing mTBI vs. sham groups (Fig. 1B, Supplementary File S1). Enrichment analysis of Biological Processes revealed 168 GO terms (*padj* < 0.05) (Supplementary File S2). Among these GO terms, 20 are related to endothelial cells or vasculature, and 6 are related to TGF-β signaling (Supplementary File S2). Pathway analysis (utilizing the Elsevier Pathway Collection) revealed vasculature/endothelia- and TGF-β signaling-related pathways as the main ones affected between mTBI and sham (Supplementary File S2). These results support the importance of vascular damage in mTBI pathophysiology, where TGF-β signaling may play a significant role. They further strengthen the rationale for testing C381 in mTBI.

Indeed, the gene expression changes induced by mTBI were largely reversed by C381 treatment, that is, genes upregulated by mTBI were downregulated by C381 treatment, and genes downregulated by mTBI were upregulated by C381 treatment (Fig. 1D). In addition, by comparing the vehicle-treated mTBI group (mTBI + veh) versus C381-treated mTBI group (mTBI + C381), we identified 64 C381-induced, differentially expressed genes (DEGs) (*padj* <0.05) (Fig. 1C, Supplementary File S3). Among the DEGs, many have been linked to TGF-β signaling (e.g., DSP,^31^ Lingo3,^32^ Npnt,^33^ Apcdd1,^34^ Lefty1,^35^ Net1,^36^ Sf3b1,^37^ Hmgcr,^38^ St3gal1,^39^ Itpka,^40^ and Cldn2^41^), and/or lysosomal function (DSP,^42^ lingo3,^43^ Calb1,^44^ Itpka,^45^ and Cldn2^46^) and are enriched in brain endothelial cells (e.g., DSP, Npnt, Apcdd1, Plekha2, Net1, and St3gal1). Many are signaling molecules involved in neuroprotection, neuroregeneration, and survival (e.g., Calb1,^44^ Npnt,^47^ Apcdd1,^48^ Lefty1,^49^ Net1,^50^ Sf3b1,^51^ lingo3,^43^ Hmgcr,^52^ St3gal1,^53^ Itpka,^54^ and Cntnap4^55^). These findings corroborate the impact of C381 on TGF-β signaling and lysosomal function while also indicating that brain endothelial cells may represent one of the target cell types affected by C381 treatment.

### Postinjury administration of C381 attenuates acute cerebral vasculature damage

**FIG. 2.**
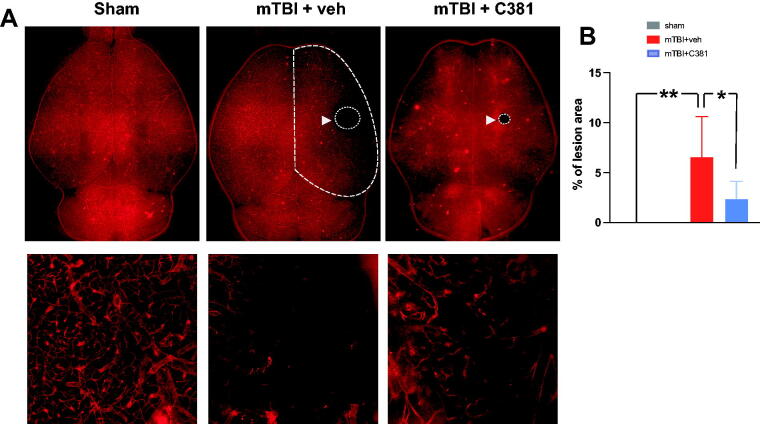
C381 treatment attenuates brain vessel damage. Vessel painting was performed 3 days after mild traumatic brain injury. **(A)** Wide field epifluorescence images of the whole brain (top panel), higher-magnification view of the injury site (bottom panel). **(B)** Lesion area was quantified as the percentage of dark area out of the hemibrain. **, *p* < 0.01; *, *p* < 0.05, by analysis of variance (ANOVA) and Bonferroni’s multiple comparison test.

**FIG. 3.**
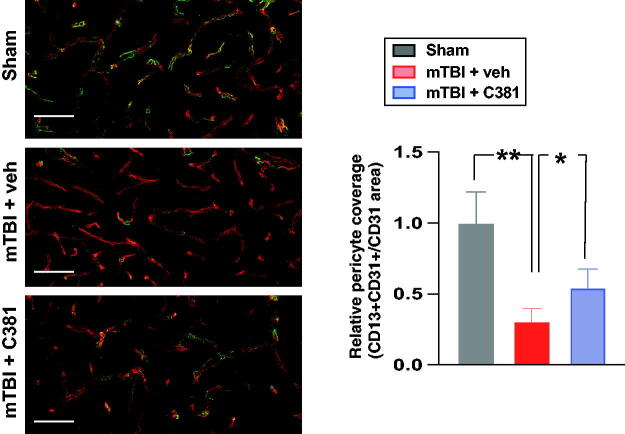
C381 treatment attenuates pericyte loss. The mice were sacrificed 3 days after mild traumatic brain injury. Brain sections were then immunostained with antibodies against CD31 and CD13. The relative coverage of CD13 in the ipsilateral hippocampus was analyzed semi-quantitatively with thresholding analysis using ImageJ software and expressed as CD13+CD31 over CD31 area. **, *p* < 0.01; *, *p* < 0.05, by analysis of variance (ANOVA) and Bonferroni’s multiple comparison test. Scale bars = 20 µm.

To test if C381 could exert protection on cerebral vasculature after mTBI, we performed vessel painting. Vessel painting with the lipophilic carbocyanine dyes 1,1′-dioctadecyl-3,3,3′,3′-tetramethylindocarbocyanine perchlorate (DiIC18) provides a rapid and reliable method to visualize blood vessels in experimental animals.^56,57^ This technique has been utilized in the examination of acute cerebrovascular response following TBI.^58–60^ Recent advancements have enhanced this method by using neutral liposomes and 1,1′-didodecyl-3,3,3′,3′-tetramethylindocarbocyanine perchlorate (DiIC12) to achieve brighter labelling of all major blood vessel types in mouse brain.^29^ Here we performed vessel painting with neutral liposomes and DiIC12 in a repetitive mTBI paradigm previously developed in our lab.^26,27^ In agreement with previous findings, vessel-painted brains showed excellent labeling of the cortical and subcortical vessels in the sham animals, with no obvious disruption of the vessels. Vehicle-treated mTBI mice showed clear vascular loss/disruption at the site of the injury; but C381-treated mice showed significant reduction of vascular damage (Fig. 2). Under higher magnification, the vessel damage after mTBI was marked by discontinuous DiIC12 labeling, vessel fragmentation, and leakage of the DiI DiIC12 (Fig. 2).mTBI-induced cerebrovascular injury is often accompanied with BBB breakdown, pericyte loss, and cerebral blood flow reduction.^15,61^ Pericyte loss contributes to BBB disruption and dysfunction following TBI^15^ and during aging.^62^ To investigate whether C381 treatment was associated with reduced pericyte loss, we performed immunohistochemistry using an antibody against CD13 to label pericytes and another one against CD31 to label endothelial cells. In agreement with previous reports,^15,61^ pericyte coverage was significantly decreased in the ipsilateral cortex in the mTBI group compared with the sham group (Fig. 3). Treatment with C381 significantly increased pericyte coverage (Fig. 3). Together, these results demonstrate that treatment with C381 exerts strong protective effects against vascular damage after mTBI.

### C381 treatment reduces astrocyte reactivity

**FIG. 4.**
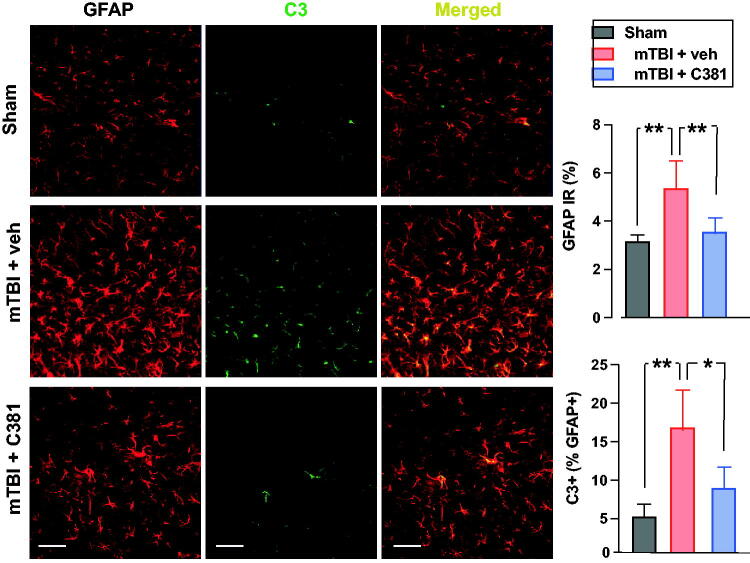
C381 treatment reduces astrocyte reactivity after mild traumatic brain injury (mTBI). The mice were sacrificed 1 month after mTBI. Brain sections were then immunostained with antibodies against GFAP and C3. The immunoreactivity of GFAP in the ipsilateral hippocampus was analyzed semi-quantitatively with thresholding analysis using ImageJ software. C3-positive astrocytes (C3+/GFAP+) were quantified as percentage of total GFAP+ astrocytes. **, *p* < 0.01; *, *p* < 0.05, by analysis of variance (ANOVA) and Bonferroni’s multiple comparison test. Scale bars = 50 µm.

Chronic astrocyte reactivity is a pathological feature of the repetitive mTBI model.^26,27^ We therefore sought to determine if C381 treatment would reduce astrocyte reactivity. Astrocyte reactivity assessed by GFAP immunoreactivity was significantly increased in the ipsilateral hippocampus of vehicle-treated mTBI animals relative to sham (Fig. 4). Treatment with C381 significantly attenuated mTBI-induced GFAP immunoreactivity relative to vehicle-treated mTBI animals (Fig. 4). In addition, complement C3 has been recently identified as a marker for reactive astrocytes.^63^ C3-positive astrocytes (assessed as percentage of total GFAP-positive astrocytes) were significantly increased after mTBI in the vehicle-treated mTBI group but were significantly decreased in the C381-treated mTBI group (Fig. 4).

### C381 treatment restores calbindin expression

**FIG. 5.**
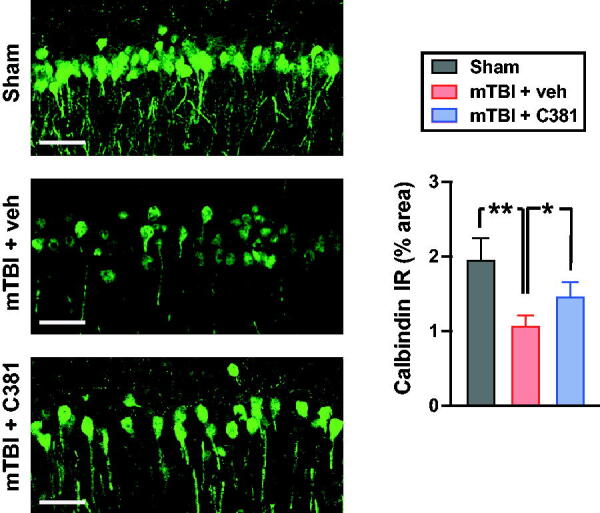
C381 treatment restores calbindin immunoreactivity after mild traumatic brain injury (mTBI). The mice were sacrificed 1 month after mTBI. Brain sections were immunostained with antibodies against calbindin. The immunoreactivity of ipsilateral hippocampus CA1 was analyzed semi-quantitatively with thresholding image analysis using ImageJ software. *, *p* < 0.05; **, *p* < 0.01, analysis of variance (ANOVA) and Bonferroni’s multiple comparison test. Scale bars = 50 µm.

Among the DEGs affected by C381 treatment, Calb1 (calbindin) has been linked to both TGF-β signaling^64,65^ and lysosomal function.^44^ To validate the expression changes of calbindin at the protein level, we performed immunohistochemistry with an anti-calbindin antibody. In the sham animals, calbindin-immunopositive neurons were observed in CA1, CA2, and dentate gyrus subregions of the hippocampus. Compared with the sham group, the number of calbindin-positive neurons and intensity of calbindin immunoreactivity were decreased in the vehicle-treated mTBI group (mTBI + veh), especially in the CA1 region (Fig. 5). C381 treatment significantly increased intensity of calbindin immunoreactivity in the C381-treated mTBI group (mTBI + C381) (Fig. 5). These results show that calbindin expression was reduced after mTBI but was largely restored by C381 treatment.

## Discussion

The findings from our study, integrating RNA-seq data with histological analysis, provide compelling evidence supporting the critical role of blood vessel damage in mTBI pathophysiology and the protective effects of the small molecule drug C381 in attenuating this damage. We show that systemic administration of C381 effectively attenuates mTBI-induced vascular damage and restores pericyte coverage in a concussive rodent model. Our results underscore the intricate relationship between blood vessel damage and TGF-β signaling in the context of TBI pathophysiology.

Our RNA-seq results revealed significant alterations in gene expression patterns associated with mTBI. We discerned a profound dysregulation in genes intricately linked to vascular integrity and function after mTBI. Noteworthy alterations encompassed the dysregulation of genes associated with endothelial dysfunction, BBB compromise, and vascular damage. As a result, the pathways related to vasculature and endothelial cells emerged as prominently affected, implicating vascular dysfunction in the aftermath of mTBI. This aligns with existing literature highlighting the susceptibility of brain vasculature to trauma-induced brain injury and the ensuing cascade of pathological events.^12–14^ Furthermore, our RNA-sequencing analysis identified TGF-β signaling-related processes and pathways as significantly affected by mTBI, emphasizing a potential intricate interplay between vascular perturbations and TGF-β signaling cascades after mTBI. Notably, the gene expression alterations triggered by mTBI were predominantly counteracted by C381 treatment, underscoring the therapeutic promise of C381 in mTBI.

Building upon these molecular insights, our histological analysis provided direct visualization of tissue-level changes, corroborating the RNA-seq findings. Specifically, we observed that C381 treatment attenuated blood vessel damage in the injured brain tissue. This observation not only validates the relevance of our molecular findings but also supports the potential therapeutic benefits of targeting vascular integrity in mTBI management.

The protective effect of C381 on blood vessels is consistent with the known role of TGF-β signaling in supporting endothelial cells,^20^ the primary cellular constituents of blood vessels. C381 was initially developed as an activator for TGF-β signaling^24^ and recognized for its trophic effects on various cell types, including endothelial cells. By bolstering endothelial cell health, C381 may mitigate the deleterious consequences of vascular injury following mTBI, ultimately contributing to tissue repair and recovery.

The RNA-seq analysis further revealed that C381 treatment largely reversed the gene expression changes induced by mTBI and many of the DEGs affected by C381 treatment are associated with TGF-β signaling and/or lysosomal function. These findings align with our previous study, demonstrating that C381 not only activates the TGF-β signaling pathway but also significantly enhances lysosomal function.^25^ Such consistency suggests the specific relevance of these pathways in C381 treatment. Calbindin (also known as calbindin 1 or calbindin-D28k) has been linked to both TGF-β signaling and lysosomal function. Deficiency of TGF-β signaling leads to reduced calbindin expression.^64,65^ Calbindin has been suggested to have a neuroprotective effect in the brain tissues and its protective effect is dependent on lysosomal function.^44^ Calbindin knockout mice show impaired spatial learning and fail to maintain long-term potentiation, suggesting a critical role of calbindin for hippocampal function and cognition.^66^ In Alzheimer’s disease model mice, the absence of calbindin exacerbated neuronal loss, triggering significant apoptotic features, and mitochondrial dysfunction.^67^ Additionally, calbindin expression is significantly decreased in various neurological disorders, including aging, neurodegenerative diseases^68^ and chronic mTBI.^69^ Therefore, restoration of calbindin signaling has the potential for mitigating brain pathology. Notably, in the context of C381 treatment, RAN-seq analysis and immunohistochemistry studies showed that C381 treatment can restore or maintain calbindin levels. Hence, the impact of C381 on calbindin may represent an underlying mechanism contributing to its efficacy in alleviating mTBI pathology.

We have previously shown that C381 mitigates gliosis, shields neurons from neurotoxins, and enhances cognitive function across various mouse models of chronic neurodegenerative diseases.^25^ These results suggest that C381 holds promise as a therapeutic agent for these conditions.^24^ The findings presented here reveal that C381 also mitigates astrogliosis and exerts neuroprotective effects, including the restoration of calbindin levels, following mTBI. Moreover, they demonstrate that C381 diminishes cerebrovascular damage. Therefore, these findings not only expand the potential clinical applicability of C381 to mTBI but also lend support to its action on the cerebrovasculature as a component of C381’s pharmacological effects in acute brain injury.

Inflammation is an important contributor to the pathology of TBI, contributing significantly to secondary injury mechanisms and influencing the outcome of the injury.^70–72^ Astrogliosis, characterized by the reactive response of astrocytes in the brain, is considered a hallmark sign of inflammation following TBI.^73^ This reactive astrogliosis involves the proliferation and hypertrophy of astrocytes, leading to the formation of a glial scar around the injury site.^73^ C381’s ability to reduce astrogliosis may be mediated through several mechanisms, one of which involves its potential to mimic the anti-inflammatory effects of TGF-β. TGF-β is a multifunctional cytokine known for its immunomodulatory properties, including the suppression of inflammatory responses and the promotion of tissue repair processes.^22^ By activating similar signaling pathways as TGF-β, C381 may exert anti-inflammatory effects within the central nervous system, thereby dampening the astrocytic response to injury or inflammation. Additionally, C381 may contribute to the reduction of astrogliosis by indirectly preserving BBB integrity. Disruption of the BBB and leakage of blood components into surrounding brain tissue can trigger inflammation and astrogliosis.^10,11^

The precise mechanism underlying the therapeutic effects of C381 remains ambiguous, raising questions about whether its impact is primarily direct on neurons or mediated through interactions with other cell types like endothelia and immune cells. While our previous study suggest a direct influence on neuronal function, they also propose that C381 may exert its benefits indirectly by modulating the activity of microglia and immune cells.^25^ This study demonstrates that the beneficial effects of C381 treatment may potentially stem from its capacity to support endothelial cells and mitigate blood vessel damage, as evidenced by both molecular and histological analyses. Further research is needed to elucidate the intricate interplay between C381 and various cellular components within the central nervous system.

In summary, our study underscores the therapeutic promise of C381 in treating mTBI. Given the absence of toxicity concerns noted in previous studies,^25^ our findings present compelling evidence advocating for the potential of C381 as a therapeutic option for mTBI. However, further investigation is warranted to fully understand its therapeutic mechanisms and optimize its clinical application. It would be valuable to explore the efficacy of C381 treatment in preclinical models of mTBI that already exhibit established vascular pathology.

## Abbreviations Used

BBB blood: brain barrier
DEG: differentially expressed genes
GFAP: Glial fibrillary acidic protein
TBI: Traumatic brain injury
TGF-β: Transforming growth factor-β
